# Schistosomiasis infections in South African pregnant women: A review

**DOI:** 10.4102/sajid.v35i1.171

**Published:** 2020-09-23

**Authors:** Melissa D. Bengu, Vinogrin Dorsamy, Jagidesa Moodley

**Affiliations:** 1 Department of Obstetrics and Gynaecology, College of Health Sciences, University of KwaZulu-Natal, Durban, South Africa; 2 Department of Laboratory Medicine and Medical Sciences, Faculty of Health Sciences, University of KwaZulu-Natal, Durban, South Africa

**Keywords:** schistosomiasis, pregnancy, parasites, praziquantel, South Africa

## Abstract

**Background:**

Schistosomiasis, a chronic parasitic disease caused by Schistosoma species, has a negative impact on pregnancy outcomes and child development. The disease affects over 230 million people worldwide, and in South Africa an estimated 5.2 million people are thought to be infected. However, there is scant data on the impact of schistosomiasis in pregnancy in South Africa and globally. The aim of this review was to analyse the current knowledge of schistosomiasis in pregnancy, particularly in South Africa, focusing on maternal and neonatal complications linked directly to the disease or its treatment.

**Methods:**

An electronic search of online databases was used to identify and collect relevant research articles related to schistosomiasis in pregnancy, with a focus on South Africa.

**Results:**

Schistosomiasis can cause severe organ damage when left untreated and influences maternal and foetal morbidity and mortality. Although South Africa’s first helminth control programme was established in 1997, there is currently no ongoing treatment strategy programme, and little information is available on prevalence rates in pregnant women for the last 20 years. There is also an absence of data from well-controlled clinical trials that focus on the efficacy and safety of treatment during pregnancy, which has led to this vulnerable group being neglected.

**Conclusion:**

This review highlights the dearth of information on the impact of schistosomiasis in pregnant women in South Africa and the need for high-quality evidence-based studies.

## Background

Schistosomiasis (bilharzia) is a common chronic disease caused by parasitic infection with trematodes of the genus *Schistosoma*. It is one of the most prevalent tropical diseases and is the second most important neglected tropical disease.^1^ In 2017, an estimated 220 million people required preventive treatment and about 700 million people were said to be at risk of infection globally.^2^ Infections are complicated by socio-demographic factors associated with poverty such as lack of access to clean water and adequate sanitation, as well as co-infections with other helminth infections, malaria, tuberculosis (TB), human immunodeficiency virus (HIV) and acquired immunodeficiency syndrome (AIDS).^3^

The disease is endemic to Africa, Asia, the Middle East and South America.^4^ Its prevalence is highest in Africa, with over 90% of those requiring treatment living on the continent.^2^ Little progress has been made in reducing disease morbidity in affected regions, despite the public awareness campaigns and policies that are in place.^5^ Some countries have even seen an increase in infection levels^6^ because of human migration to urban areas. This, coupled with the increased ease of mobility, poses a risk of re-establishing *Schistosoma* species in areas that are non-endemic, or where the disease has been eliminated.^7^

Schistosomiasis is endemic to some regions of South Africa (SA).^8^ Research on prevalence has focused largely on children, and treatment strategies aim at eradicating the disease in this vulnerable group.^9^ This is not without warrant. Children play the largest role in local disease transmission, and more importantly, the effects of infection have significant immediate and temporal consequences. Without treatment, infection leads to malnutrition and anaemia, retarded growth and poor performance in school.^9,10^ Chronic infection is associated with sustained malnutritive disorders that affect their growth and cognitive development^10^ and causes chronic inflammation of the organs, which can lead to death in severe cases. Such longstanding infection and reinfection in endemic areas translates into morbidity in adults.^6^ When the body responds to parasites embedded in the tissue, this can lead to collateral damage of the tissue and may translate into urogenital disorders in adulthood.^11^

While research on schistosomiasis in children is critical, other vulnerable groups should also be considered. These children will become adults, half of whom will fall pregnant. Research on the prevalence or treatment in women of reproductive age and during pregnancy is scarce globally, and equally true in SA.^5,12^ Studies conducted locally have focused on isolated endemic areas and are largely conducted on school-aged children with virtually none on pregnant women.^13^

Although women of reproductive age are at less risk to infection than their male counterparts, chronic exposure and infection may lead to overt and long-term sequelae during and after pregnancy.^14^ Pregnancy naturally increases physiologic demand of most organ systems, and a successful pregnancy outcome is governed by the health status of the mother even prior to pregnancy.^15^ Schistosomiasis further affects the uterine environment, besides its contribution to anaemia and malnutrition.^16^ Infected women have an increased risk for ectopic pregnancies and higher rate of spontaneous miscarriage.^17^ When coupled with poor living conditions, it increases a mother’s risk of mortality and is directly related to premature birth, low birth weight and morbidity of infants.^12^ This translates into poor growth and development during early childhood, and increased childhood risk for schistosomiasis in endemic areas, thereby compounding the burden.^11^

While most mitigation efforts focus on morbidity control and elimination strategies in children, an awareness and implementation of treatment strategies is of vital importance in pregnant women as well. The World Health Organization (WHO) has proposed prophylactic chemotherapy as a treatment strategy that focuses on reducing disease through regular, targeted treatment of affected populations.^2^ At present, praziquantel (PZQ) is the treatment of choice for all schistosome infections, as it is effective and inexpensive and has been used successfully for > 30 years.^18^ Treatment is of paramount importance because if infection is left untreated, normal physiological functions such as iron metabolism, physical fitness and cognitive function are impaired, resulting in systemic morbidities such as anaemia, malnutrition as well as impaired development in children.^11,19^ Schistosomiasis also occurs alongside other infectious diseases, affecting immunological and physiological relations between the host and co-infecting pathogens.^20^ While this may be tolerated in non-pregnant women, successful pregnancy is dependent on adapting the immune system to accommodate a semi-allogeneic foetus, and schistosomiasis alters this dynamic.^21^ Thus, better control of schistosomiasis in women of reproductive age and during pregnancy results in better pregnancy outcomes for both mother and child, with additional benefits of control of other diseases.^22^

The objective of this review is to summarise the existing literature on schistosomiasis in pregnant women and to identify research gaps relating to schistosomiasis, particularly in SA.

### Schistosome life cycle in humans

**FIGURE 1:**
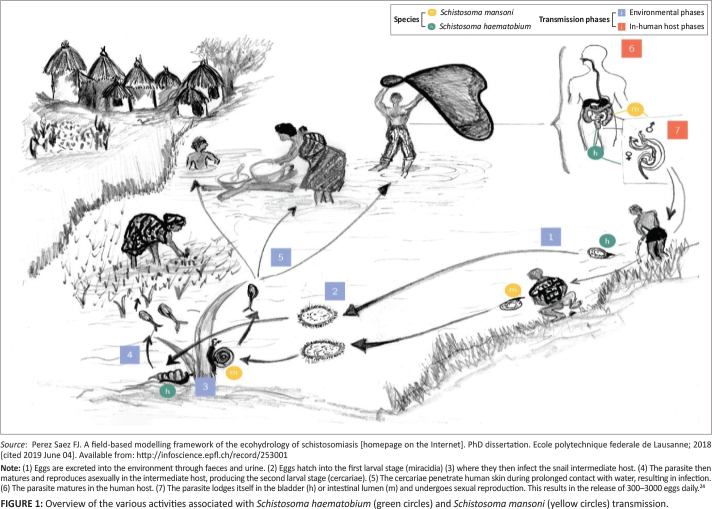
Overview of the various activities associated with *Schistosoma haematobium* (green circles) and *Schistosoma mansoni* (yellow circles) transmission.

There are three main species of *Schistosoma* that infect humans, *Schistosoma haematobium, Schistosoma mansoni* and *Schistosoma japonicum*.^23^ Of these, *S. haematobium* and *S. mansoni* occur mainly in Africa and the Middle East, whereas only *S. mansoni* is present in South America and *S. japonicum* is localised to Asia.^2,4,11,23^ In geographical areas where schistosomes are endemic, infection in humans happens during routine agricultural, domestic, occupational and recreational activities, and are associated with a lack of clean water and inadequate sanitation. Figure 1^24^ shows the transmission of *S. haematobium* and *S. mansoni*. Adult schistosomes have an average intra-host lifespan of 5–10 years which allows the parasites to remain in blood vessels producing eggs, developing into chronic infection.^2,11^ While the adult schistosomes develop the ability to mask against a host immunogenic response, the eggs of *S. mansoni* and *S. japonicum* or *S. haematobium*, when migrating through intestinal submucosa or the bladder wall, respectively, elicit an inflammatory response that ultimately results in scarring and fibrosis in these organ systems.^2^ Eggs released into the bloodstream can also affect other organ systems such as the liver, lungs and the brain.^11^

## Review methodology

An electronic search of the following databases was conducted: PubMed/MEDLINE, Google Scholar and Scopus. The search terms included schistosomiasis, bilharzia, pregnancy, pregnant, gestation, women and South Africa. Boolean terms (AND, OR) were used to separate the keywords, and Medical Subject Headings (MESH) terms were included during the search. Websites such as the WHO and governmental websites were searched for policies and guidelines regarding diagnosis and management of anaemia in pregnancy. Relevant studies were identified by searching literature from January 1985 to date. Articles were also searched through the ‘Cited by’ search as well as citations included in the reference lists of included articles.

The search strategy was piloted to check the appropriateness of selected electronic databases and key words. A PubMed search using MeSH terms ‘Schistosomiasis’ and ‘South Africa’ and filtered for studies involving humans returned 495 articles with no restrictions on date of publication. Eligibility criteria for selection of articles included being available in full text and related to schistosomiasis in pregnancy, and involve South African pregnant women.

An initial title screening reduced the number of articles to 76, and from this pool, relevant articles were gleaned for necessary information.

## Review findings

### Schistosomiasis in South Africa: Historical data

Schistosomiasis is endemic to SA and poses a challenge to public health. According to the WHO (Table 1)^25^ by 2011, an estimated 5.2 million people required prophylactic drug therapy for schistosomiasis in SA.^23^

**TABLE 1:** Estimates of the South African population requiring preventive chemotherapy for schistosomiasis.

Year	SAC population requiring PC for SCH annually	Population requiring PC for SCH annually
2010	2 438 847	5 190 811
2011	2 444 487	5 220 200
2012	2 457 968	5 248 988
2013	2 476 276	5 288 087
2014	2 493 327	5 324 499
2015	2 517 413	5 375 934
2016	2 550 388	5 446 352
2017	2 575 127	5 499 182

*Source:* World Health Organization. Schistosomiasis: Population requiring preventive chemotherapy and number of people treated in 2010. Releve Epidemiol Hebdomadaire. 2012;87(4):37–44.

PC, preventive chemotherapy; SAC, school age children; SCH, schistosomiasis.

Note: SAC population requiring PC for SCH annually: estimated number of school age children requiring preventive chemotherapy for schistosomiasis annually according to the recommended strategy. Population requiring PC for SCH annually: estimated number of individuals requiring preventive chemotherapy for schistosomiasis annually according to the recommended strategy.

**FIGURE 2:**
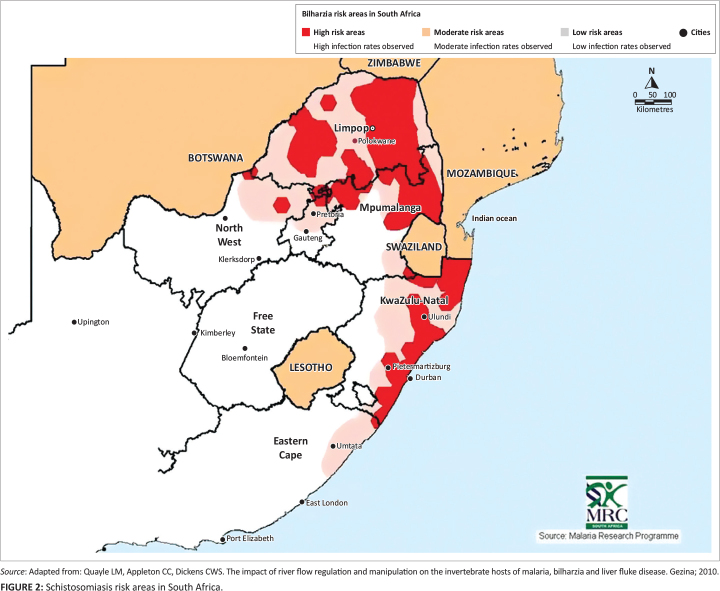
Schistosomiasis risk areas in South Africa.

While some studies on schistosomiasis prevalence rates were undertaken in endemic regions of SA (depicted in Figure 2)^30^ in the 1980s and 1990s,^26,27^ there is a dearth of information available for the last 20 years.^28^ However, in 2012, an increase in disease occurrence was noted in all nine provinces, including the Northern Cape which was previously regarded as a non-transmission region.^29^ Schistosomiasis’ prevalence rates are reported to be highest in KwaZulu-Natal (KZN), Mpumalanga (MP) and Limpopo (LP) provinces,^8,30^ and in 2018, endemic areas included KZN, MP, LP, eastern and northern parts of Gauteng province as well as coastal areas of the Eastern Cape.^31^

### Schistosomiasis in pregnancy

Pregnant women, particularly those living in low- and middle-income countries (LMICs), are at increased risk for significant maternal and foetal morbidity and mortality, because of multiple contributing factors.^5,12^ Keys amongst these are helminth infections, which are associated with a lower quality of life in women during and after pregnancy, as helminth infections can lead to anaemia, changes in host immune responses, nutritional deficiencies and other more serious complications, such as end organ damage.^15^ Altered host immunity during pregnancy increases susceptibility to disease and increases transmission risk for helminth infections,^32^ which, in turn, increases the risk of co-infection with other diseases and adversely affects progression of disease, worsening its outcomes.^33^ In sub-Saharan Africa, this contributes to the HIV/AIDS and TB epidemics, which increases maternal mortality and the likelihood of poor neonatal outcomes.^34,35,36^

Schistosomiasis causes an array of adverse outcomes in pregnant women.^33^ It interferes with nutrient uptake impairing the nutritional status of the infected mother and causing malnutrition which leads to anaemia as the nutrients that are essential for blood cell formation become reduced or depleted.^32^ Chronic schistosomiasis is believed to cause anaemia as a result of effects of pro-inflammatory cytokines produced in response to infection and decreased iron bioavailability because of upregulation of hepcidin in response to a *Schistosoma*-associated cytokine.^37^

Extracorporeal blood loss when eggs are shed through the gut wall (*S. mansoni and S. japonicum*) or through repeat inflammation of the bladder wall and urethra (*S. haematobium*) causes iron deficiency.^11^ Socio-economic factors, coupled with subsequent malnutrition because of Schistosoma infection and possible co-infections, lead to iron deficiency anaemia in women of reproductive age and are exacerbated in pregnant women.^38^ Anaemia is a major risk factor for preterm labour, low birthweight, stillbirth and maternal death,^12^ and there is a positive correlation between anaemia and infection intensity.^39^ In heavily infected pregnant women, the morbidity associated with anaemia is more prominent.^5^

Schistosomiasis can cause maternal, placental and foetal infection.^40^ Infection with *S. haematobium* is associated with increased pro-inflammatory responses amongst circulating leucocytes^41^ which can cause inflammation of the cervix leading to spontaneous miscarriage or of the fallopian tubes resulting in ectopic pregnancy.^17^ *Schistosoma mansoni* is associated with elevated concentrations of circulating endotoxins.^42^ When found in high concentration in the placenta, these endotoxins have been associated with placental inflammation and preterm labour.^43^ *Schistosoma japonicum* is associated with an increase in systemic inflammatory mediators.^44^ This causes a pro-inflammatory state in the foetal and maternal compartments and has been associated with low birthweight.^40^ These studies make the link between maternal schistosomiasis and adverse birth outcomes evident and highlight the need for treatment of schistosomiasis during pregnancy.

Schistosomiasis also impacts future pregnancies, and *S. haematobium* in non-pregnant women may cause female genital schistosomiasis (FGS).^22^ Galappaththi-Arachchige et al. reported that in areas where *S. haematobium* is endemic, FGS is a neglected cause of reproductive morbidity.^45^ Female genital schistosomiasis affects the reproductive tract and is characterised by the presence of schistosome eggs or worms in the epithelium of the urinary bladder, female genital organs or genital mucosa.^8^ It is associated with ectopic pregnancies, genital symptoms that are similar to those of sexually transmitted diseases (STDs), infertility and miscarriage, and studies have shown that it may make women more susceptible to HIV.^45^

Despite the many case reports and studies that have shown an association of female genital tract infection with schistosomiasis in pregnant populations,^5,46^ there is relatively limited literature that specifically focuses on schistosomiasis and subsequent birth outcomes.^47,48^ There has also been no data presented on maternal schistosomiasis in countries that have high paediatric schistosomiasis.^5^

Colley et al. reported that globally, *S. haematobium, S. mansoni* and *S. japonicum* infected an estimated 40 million women of childbearing age.^11^ Global estimates by the WHO have also shown that annually, over 10 million women in Africa contract schistosomiasis during pregnancy.^23^ Despite the high prevalence of this important condition, there are still gaps in the knowledge on the specific morbidity and outcomes of the disease on pregnant women and their offspring.^12^

### Maternal schistosomiasis in South Africa

Despite the large risk they present to both mother and child, very limited epidemiological data on schistosomiasis are available for the reproductive phase of life in SA. Most interventions and research conducted in SA focus on school children,^9^ justified by the highest incidence and intensity of infection amongst this 6–20-year-old group and the reported effects on their growth, physical development and school performance.^49^ Pregnant women who are exposed to the same environment are not given priority, because of concerns of treatment during gestation and lactation.^50^ This lack of research impacts on effective and timeous management of the disease, resulting in these mothers becoming infection reservoirs and making reinfection inevitable.^6^ Higher rates of teenage pregnancy conflate incidence rates with socio-economic factors and increase the impact of schistosomiasis on women of reproductive age, which is left largely untreated or ignored. This is evident in South African studies on women of childbearing age in rural areas. A study by Kleppa et al. reported a 20% prevalence rate of urogenital schistosomiasis in high school students above 16 years of age.^51^ In addition, Galappaththi-Arachchige et al. have reported 19.7% and 17.3% prevalence rates in women of childbearing age.^45,52^

There is little evidence of the effects of schistosomiasis in pregnant women in SA. A recent study showed a prevalence of *S. haematobium* of at least 17% in sexually active schoolgirls aged 16–22 in a rural endemic area of KZN.^52^ The study compared various diagnostic test sensitivities for detecting active and past exposure. Notable urogenital lesions were present in the study population which could possibly be misdiagnosed as a sexually transmitted infection. These lesions cause genital itching, bleeding and dyspareunia and may lead to infertility, and continue to manifest even though ovum laying worms are no longer present and likely to affect reproductive health.^48^

### Current needs

Schistosomiasis can be treated, and snail reduction has shown to benefit endemic populations.^53^ Early and effective treatment can benefit pregnant women.^54^ However, the lack of data from well-controlled clinical trials that focus on the efficacy and safety of PZQ during pregnancy, compounded by previous recommendations that treatment should be avoided during pregnancy and lactation, has led to pregnant women being excluded from treatment.^54^ When left untreated, schistosomiasis infection results in significant morbidity and mortality. No high-quality epidemiological studies that have assessed the impact of schistosomiasis on pregnancy have been undertaken to date, particularly in SA.^12^

### Present strategies to reduce schistosomiasis

Currently, PZQ is the only drug recommended for the regular treatment of schistosomiasis,^19^ and when taken at a dose of 40 mg/kg,^55^ adult schistosomes are eradicated. Although the cost makes mass treatment programmes effective, at only ± R7.00 per treatment course per person,^56^ such programmes are not being implemented and the disease remains endemic in at least five of the nine provinces.^32^ This is partially because of poor education and information dissemination amongst populations at risk of infection, the lack of resources that would support PZQ distribution and delivery of the drug to areas where it is needed.^19^ The Medicines Control Council and the National Department of Health have also only approved brand name drugs (such as Bayer’s Biltricide^®^) for treatment, while having stringent regulation in place that makes manufacturing generic PZQ a costly and time-consuming process, thereby making treatment unaffordable in afflicted districts.^57^

## Implications

The lack of sufficient, well-controlled studies on the use of PZQ in pregnant and lactating women has deterred many countries (including SA) from using this drug during pregnancy.^9,58^ As a result, implementing treatment strategies has been inconsistent^32^ despite WHO recommendations that, in endemic areas, pregnant and lactating women be treated with PZQ^59^ and that anti-helminth treatment be incorporated into antenatal care after the first trimester of pregnancy.^60^ This is despite there being no severe adverse incidents reported in non-interventional studies where pregnant women were treated or accidentally exposed to PZQ.^58^ These reports include a Sudanese study in which 88 women were exposed to PZQ (37 during the first trimester of their pregnancy). None of the pregnancies resulted in stillbirths or miscarriages, and there were no congenital abnormalities.^61^ Praziquantel was found to have no significant effect on birth weight, congenital anomalies, maternal anaemia or perinatal mortality in a randomised, double-blind, placebo-controlled trial that recruited 2507 pregnant women to investigate the benefits of anthelminthic use during pregnancy.^62^ Thus, there certainly may be a place for the treatment of schistosomiasis in the second and third trimesters of pregnancy. In addition, healthcare professionals should investigate for schistosomiasis in pregnant women presenting with haematuria, and persistent or recurrent urinary tract infections, especially in HIV-positive women.^58^ To effectively control schistosomiasis, early detection of the disease is required to ensure optimum efficiency of both control and treatment programmes.^19,29^ In sub-Saharan Africa, the incidence of infection ranges from 2.5% to 63.5% in maternal schistosomiasis, and this will gradually increase with time if the recommended interventions and preventative measures are not implemented.^5^

## Conclusion

Schistosomiasis is a public health concern in SA with approximately a tenth of the population at risk. There is significant evidence implicating schistosomiasis directly in maternal and foetal morbidity and mortality, and indirectly by causing nutrient deficiencies leading to anaemia and malnutrition or by association with other co-morbidities such as HIV and TB. However, very little data exist on schistosomiasis amongst pregnant women in SA. Research on the distribution and prevalence of schistosomiasis, as well as frequent and consistent surveillance and intervention programmes that target all population groups, especially pregnant women, will contribute significantly to controlling this disease.^29,55^

High-quality evidence-based studies assessing the impact of schistosomiasis on pregnancy are urgently required to support global intervention and treatment programmes in pregnant and lactating women and their babies.
